# Homeobox B9 Promotes the Progression of Hepatocellular Carcinoma via TGF-*β*1/Smad and ERK1/2 Signaling Pathways

**DOI:** 10.1155/2022/1080315

**Published:** 2022-09-16

**Authors:** Lizhi Bai, Pan Ge, Yanni Zhang, Yi Song, Rong Xing, Dangxia Zhou

**Affiliations:** ^1^Department of Pathology, Health Science Center, Xi'an Jiaotong University, Xi'an, China; ^2^Department of Emergency, Zhongshan Hospital of Dalian University, Dalian, China; ^3^Department of Pathology and Pathophysiology, Dalian Medical University, Dalian, China

## Abstract

**Objectives:**

Homeobox B9 (HOXB9), a homeodomain-containing transcription factor, may play a role in hepatocellular carcinoma (HCC) progression. However, the exact mechanisms underlying its action remain unclear. *Materials and methods*. Immunohistochemistry was used to investigate the expression of HOBX9 and its prognostic values in HCC patients. HCC cells were transfected with pBabe-HOXB9 and shHOXB9 plasmids, and MTT assay, Transwell assays, and xenograft mouse models were employed to determine the effects of HOXB9 on HCC cell proliferation, migration, and invasion *in vitro* and *in vivo*. The biological mechanisms involved in the role of HOXB9 were determined with Western blot and RT-qPCR methods.

**Results:**

HOXB9 expression was significantly increased in HCC tissues and cell lines. Patients with higher HOXB9 levels were associated with poor prognosis. Overexpression of HOXB9 in BEL-7405 cells promoted proliferation, migration, and invasion, whereas knockdown of HOXB9 in HepG2 cells significantly reduced cell proliferation, migration, and invasion abilities. Mechanically, a positive correlation was found between HOXB9 expression and transforming growth factor-*β*1 (TGF-*β*1) and extracellular signal-regulated kinase (ERK)1/2 pathway in HCC tissues. HOXB9 overexpression stimulated TGF-*β*1/Smads signaling pathway in BEL-7405 cells. In contrast, HOXB9 knockdown inhibited the TGF-*β*1/Smads signaling pathway in HepG2 cells. In addition, the treatment with TGF-*β*1 inhibitor, LY364947, significantly reserved HOXB9 overexpression-induced cell proliferation, migration, and invasion abilities.

**Conclusions:**

These findings validated that HOXB9 promoted proliferation, migration, and invasion in HCC cells by stimulating the TGF-*β*1/Smads and ERK1/2 signaling pathway. HOXB9 could be a promising prognostic biomarker and a potential therapeutic target in HCC.

## 1. Introduction

Hepatocellular carcinoma (HCC) is the most prevalent primary liver cancer that has emerged as a critical global medical problem [1]. HCC can be caused by hepatitis B virus infection and cirrhosis [2]. Although substantial efforts have attempted to improve the prognosis of HCC, the 5-year survival rate is still less than 20% [3], and a better understanding of its molecular mechanisms is urgently needed. Tumorigenesis is mainly caused by the abnormal initiation of oncogenes, a mechanism that is equally important for the development of HCC progression [4]. The discovery of these genes associated with HCC progression makes them potentially applicable in the treatment of HCC in the future.

The homologous cassette gene HOX, a highly conserved gene family, acts to control cell proliferation and differentiation [5]. Recent studies have revealed that HOX not only plays a key role in growth and development, but is also closely related to the development of a variety of diseases, especially the process of tumor development [6]. HOXB9 is a member of the HOX gene family that regulates physiological processes such as cell proliferation, cell cycle and differentiation, embryonic segmentation, limb formation, and angiogenesis [7]. It has been found that HOXB9 could enhance the ability of lung cancer cells by regulating the proliferation and invasion through regulating epithelial-to-mesenchymal transition [8]. In breast cancer, high expression of HOXB9 stimulates the expression of various vascular factors (such as transforming growth factor, TGF-*β*) that prepares tumor cells for proliferation and metastasis [9]. In HCC, Xiong et al. found that elevated HOXB9 were closely related to tumor invasion and metastasis [10]. On the contrary, HOXB9 was also reported to be served as a tumor suppressor gene in some cancer types, and elevated HOXB9 levels were found to predict good overall survival in colon cancer [11] and gastric cancer [12]. They found that HOXB9 acetylation may account for its inhibitory role in cancer cells [13, 14]. Therefore, the role of HOXB9 in cancers may be more complicated than we assumed. However, the mechanism of HOXB9 in the generation and progression of HCC remains largely unknown.

In our previous study, we found that the activation of transforming growth factor-*β* 1 (TGF-*β*1) contributes to HOXB9-induced proliferation activities in HCC [15]. Here, we further indicated that HOXB9 contributed to the HCC tumor growth by promoting cell proliferation, migration, and invasion through TGF-*β*1-mediated Smad2 signaling and extracellular signal-related kinases (ERK1/2) signaling pathway. The present study validated that HOXB9 may serve as an oncogene to promote HCC progression and be applied as an effective therapeutic target.

## 2. Materials and Methods

### 2.1. Patient Tissue Samples

A total of 89 HCC patients, who received the curative surgery treatment in our hospital between January 2017 and December 2018, were collected. HCC tumor tissue and paired adjacent tissue were obtained from HCC patients who underwent surgical resection at the First Affiliated Hospital of Dalian Medical University and Zhongshan Hospital of Dalian University. The tissues removed surgically were liquid nitrogen and transported to our laboratory and stored at −80°C. The clinicopathological data and survival information of included patients were collected. The study was approved by the Ethics Committee of the First Affiliated Hospital of Dalian Medical University and Zhongshan Hospital of Dalian University, and each participant signed informed consents before sample collection.

### 2.2. Immunohistochemistry (IHC)

Five *μ*m sections were obtained from paraffin-embedded HCC and nontumor specimens. The sliced specimens were placed in 3% H_2_O_2_ to eliminate the activity of endogenous peroxidase after dewaxing, hydration, and antigen retrieval. Then, the sections were rinsed with PBS and incubated with an anti-HOXB9 (1 : 600; ab65063, Abcam) or anti-phosphorylated-Smad2 (1 : 1000; ab53100, Abcam) at 4°C following the manufacturer's instructions. The sections were then incubated with a goat anti-mouse IgG (H + L) secondary antibody (1: 5000; ab205719, Abcam) for 30 min at room temperature. After color development with diaminobenzidine, the sections were counterstained with hematoxylin. According to previous studies [16], the IHC score of HOXB9 depends on the intensity of staining in cells and the degree of staining. The intensity (0 = genitive, 1 = weak, 2 = moderate, and 3 = strong) and the degree of staining 1 (0-4% staining), 2 (5–19% staining), 3 (20–39% staining), 4 (40–59% staining), 5 (60–79% staining), or 6 (80–100% staining) were recorded. The final IHC score was calculated by multiplying the intensity score by the degree of staining.

### 2.3. Cell Culture

The human hepatocellular carcinoma cell line (BEL-7402, BEL-7404, BEL-7405, HepG2, Hep3B, and SNU475) and the human normal liver cell line THLE-3 all were obtained from the Cell Bank of the Type Culture Preservation Committee of the Chinese Academy of Sciences (Shanghai, China) and cultured in Dulbecco's modified Eagle's medium (DMEM) with glucose (4.5 g/l, Hyclone Laboratories, Logan, UT) supplemented with 10% fetal bovine serum (Gibco, Grand Island, NY, USA) and were maintained at 37°C and 5% CO2. BEL-7405 and HepG2 cells were then chosen to investigate the underlying mechanisms of HOXB9 in HCC because of their high expression of HOXB9 and TGF-*β*1 expression.

### 2.4. Cell Transfection

The pBabe-puro HOXB9 and pBabe-puro empty vector were purchased from Addgene and confirmed by Sanger sequencing. pBabe-puro HOXB9 was created by site directed mutagenesis of pBabe-puro HOXB9 using the Q5 site-directed mutagenesis kit (NE BioLabs) according to the manufacturer's protocol. The primers are 5′-CGCGGTACCAGACAACAATGAGAACCTTCAGGAGA-3′ (sense); 5′-GGCGAATTCCTGGCGCCGGTTACAGAACCA-3′ (antisense). Human HOXB9 (NM_024017.5) mRNA sequences were obtained from NCBI Genbank. 3 pairs of HOXB9-shRNA oligos were designed by GenScript Corporation (NJ, USA), annealed, and inserted into the pSuper-EGFP1 vector. The oligo sequence of HOXB9 was 5′-TCGACGCGAATCTCTCTTTGGCAAGTTCAAGAGACTTGCCAAAGAGAGATTCGTTTTTTGGAAT-3′ (sense); 5′-CTAGATTCCAAAAAACGAATCTCTCTTTGGCAAGTCTCTTGAACTTGCCAAAGAGAGATTCGCG-3′ (antisense). Scramble shRNA was inserted into pSuper vector (without EGFP) as control. Cell transfection was conducted using Lipofectamine 2000 (Invitrogen, CA, USA).

### 2.5. Cell Viability Assays

Cell viability was assessed using a MTT assay (Beyotime, Jiangsu, China). HepG2-Super, HepG2-shHOXB9 and BEL-7405-pBabe, BEL-7405-HOXB9 cells were seeded in 96-well plates at 5 × 10^3^ cells/well. After incubation for 0~5 days, 100 *μ*l DMEM medium containing 0.5 mg/ml MTT was added to each well for another 4 h at 37°C. The absorbance was measured at 570 nm on a microplate reader.

### 2.6. Clone Formation Assay

A total of 10^3^ HepG2-Super, HepG2-shHOXB9 and BEL-7405-pBabe, BEL-7405-HOXB9 cells were seeded in six-well plates. After 2 weeks of incubation, they were rinsed twice with PBS, fixed with 4% paraformaldehyde, and then stained with 0.5% crystal violet for 15 min and counted under the microscope.

### 2.7. Transwell Assay

The migration and invasion capacity of HCC cells were assessed through Transwell chambers (Corning Inc., Corning, NY, USA). Approximately 1 × 105 cells were plated into the upper chamber wells for migration assay, and the cells were plated into the upper chamber wells enveloped with matrigel (Corning Inc.) for invasion assay. The lower chamber was replenished with DMEM medium (500 *μ*l) containing 10% FBS. After incubation at 37°C for 24 h, the cells of the upper chamber were wiped, and the cells passed across to the membranes were fixed with paraformaldehyde and manually counted under a microscope.

### 2.8. Flow Cytometry (FCM)

After treatment, the cells were harvested for cell cycle distribution analysis in flow cytometry. Cells in differential groups were washed with PBS three times, fixed with precooled 70% ethanol, and then stained with propidium iodide (Solarbio, Beijing, China) following the protocol of the manufacturer. Finally, the stained cells were detected by a flow cytometry (Beckman Coulter, Fullerton, CA, USA) with the CellQuest software.

### 2.9. Real-Time Quantitative PCR

The total RNA was extracted from pulmonary tissues or cells by RNAiso Plus (Takara, Tokyo, Japan). cDNA with the synthesized with PrimeScript® RT reagent kit (Takara) and qPCR was performed using the SYBR Premix Ex Taq II (Takara) on the applied Biosystems 7500 Real-Time PCR System. The HOXB9 expression was normalized to GAPDH levels. Standard curves were generated, and the 2-*ΔΔ*Ct method was used to determine the expression of target genes. The PCR primers for HOXB9 were 5′-CCGGCTACGGGGACAATAA-3′ (forward); 5′-GGTGTAGGGACAGCGCTTTTT-3′ (reverse); those for GAPDH were 5′-TGCCTCCTGCACCACCAACT-3′ (forward); 5′-CCCGTTCAGCTCAGGGATGA-3′ (reverse).

### 2.10. Western Blot

Radio immunoprecipitation assay (RIPA) protein lysis buffer (Beyotime) supplemented with protease inhibitor cocktail (Roche, Basel, Switzerland) was used for protein extraction from HCC cells and HCC tissues. Protein concentration was analyzed using a BCA protein assay kit (Beyotime) following the manufacturer's protocol and then separated using 8-12% SDS-PAGE, transferred onto 0.45 mm polyvinylidene fluoride membranes (Millipore, IPVH00010, USA), and blocked with 5% skimmed milk diluted in TBS. Membranes were then incubated with the primary antibodies against HOXB9 (ab65063), Smad7 (ab227309), Smurf2 (ab53316), TGF-*β*1 (ab27969), Smad2 (ab40855), p-Smad2 (ab53100), JNK1/2 (ab199380), p-JNK1/2 (ab124956), p38 (ab170099), p-p38 (ab31828), ERK1/2 (ab17942), p-ERK1/2 (ab214362), and *β*-actin antibody (sc-47778, 1 : 1000; Santa Cruz) overnight at 4°C. After incubation with IgG (H + L) (HRP-labeled Goat Anti-Mouse IgG (Beyotime), bands were visualized with enhanced chemiluminescence (Millipore) for 2 h at 25°C. The relative band intensities were visualized with a BeyoECL Plus kit (Beyotime) and quantified using a ChemiDoc XRS+ software (Bio-Rad, USA).

### 2.11. Xenograft Model in Mice

Female BALB/c nude mice (4-6 weeks of age, weighing 16-20 g) were purchased from SLAC Animal (Shanghai, China), housed under pathogen-free conditions, and randomly divided into the pBabe group, the HOXB9 group, the pSuper group, and shHOXB9 group (5 per group). The nude mice were subcutaneously injected with Bel-7405-pBabe, Bel-7405-HOXB9 or HepG-Super, HepG-shHOXB9 cells (2 × 10^7^ in 0.5 mL PBS/mouse) on the left upper flank region of nude mice. The tumor volume was calculated using the following formula (*L* × *W*^2^)/2 (*L* is the length, and *W* is the width of the tumor). After 4 weeks following implantation, mice were all sacrificed, and the tumors were excised, weighed, photographed, and harvested for subsequent RT-qPCR, Western blot, and IHC analysis. All animal experiments were carried out according to the protocols approved by the Medical Experimental Animal Care Commission of First Affiliated Hospital of Dalian Medical University and were approved by the Animal Ethics Committee of the Dalian Medical University.

### 2.12. Statistical Analysis

Statistical analyses were performed using SPSS statistical software version 23.0 and GraphPad Prism software 7.5. The percent survival was estimated by Kaplan-Meier method. All statistical data are represented as the mean ± standard deviation. One-way analysis of variance (ANOVA) and Student's *t*-test were used for pairwise comparisons. If *P* < 0.05, the difference was considered statistically significant.

## 3. Results

### 3.1. HOXB9 Is Upregulated and Correlated with Unfavorable Prognosis in HCC

**Figure 1:**
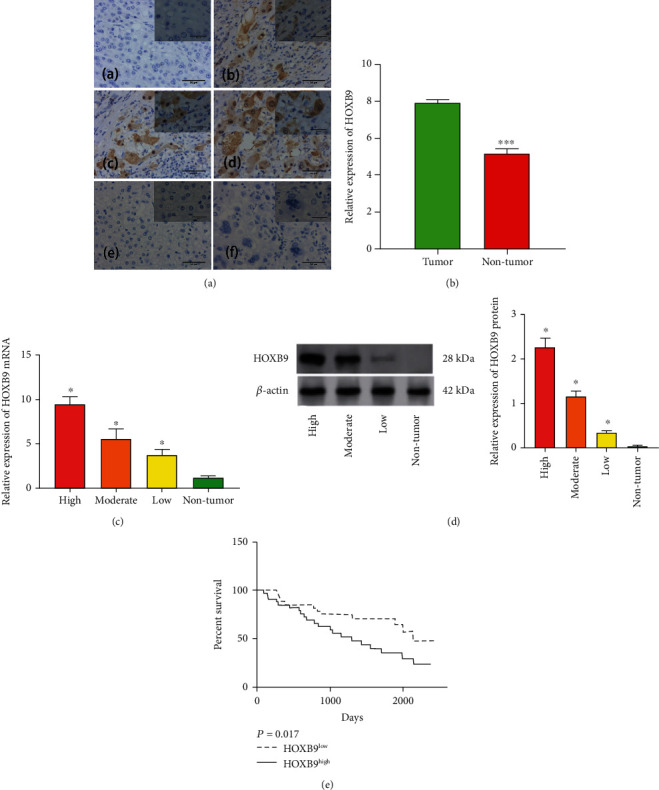
The expression profile of HOXB9 in HCC tissues. (A) Immunohistochemistry was performed to measure the expression of HOBX9 in HCC and liver tissues (200× magnification for the whole picture and 400× magnification for that at top right corner); (a) HOBX9 in nontumor tissues; (b) low HOBX9 expression in HCC tissues; (c) moderate HOBX9 expression in HCC tissues; (d) high HOBX9 expression in HCC tissues; (e–f) negative control for nontumor tissues and HCC tissues, respectively. (B) Semiquantitative analysis of HOBX9 by Immunohistochemistry in tissues from HCC tumor and nontumor. (C) RT-qPCR was used to detect the HOXB9 mRNA levels in four groups. (D) Western blot was used to detect the HOXB9 protein levels in four groups. (E) The present survival was determined in HCC patients who had HOBX9 high (*n* = 58) and low expression (*n* = 31). ^∗^*P* < 0.05 vs. nontumor.

We firstly determined the expression of HXOB9 in HCC tissues and nontumor tissues by using IHC assay. As shown in Figure 1(a), HXOB9 is located both in the nucleus and cytoplasm of HCC cells. By using IHC score, we separated the tumor tissues into three groups, including high, moderate, and low group. HXOB9 was highly expressed in the HCC tissues when compared with normal tissues (Figure 1(b)). Subsequent RT-qPCR and Western blot analysis showed that the expression of HOXB9 in the low, moderate, and high group was upregulated gradually when comparable to those in the nontumor group (Figures 1(c) and 1(d)).

We further explore the association between the HXOB9 levels and clinical characteristics. Due to the relatively small samples, we assigned the patients with moderate and low HOXB9 as one group. As illustrated in Table 1, HOXB9 is positively correlated with tumor size (*P* = 0.028). Nevertheless, HXOB9 levels were not associated with other clinical characteristics, containing age, gender, histological differentiation, liver cirrhosis, metastasis, recurrence, HBsAg status, and serum AFP levels (*P* > 0.05 for all). In addition, Kaplan-Meier survival analysis then showed poorer percent survival of HCC patients with high HXOB9 levels (Figure 1(e)). The above data showed that increased HOBX9 in HCC indicated poor prognosis.

### 3.2. Correlation of HOXB9 and TGF-*β*1 Signaling Pathway in HCC Samples and Cell Lines

**Figure 2:**
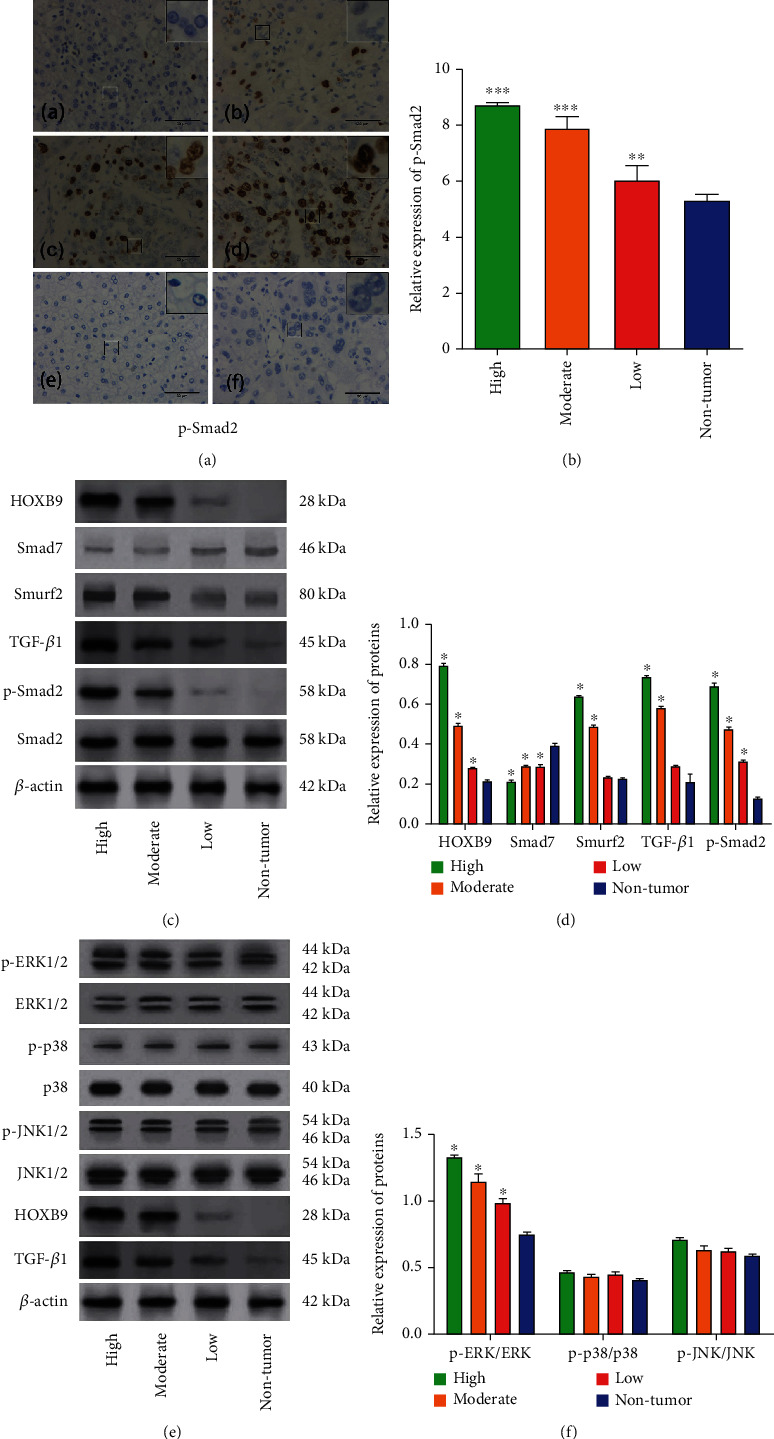
Expression prolife of TGF-*β*1 pathway and MAPK pathway in different HOXB9 expressed HCC tissues. (A) Immunohistochemistry was performed to measure the expression of p-Smad2 in different HOXB9 expressed HCC tissues (200× magnification for the whole picture and 400× magnification for that at top right corner). (a) p-Smad2 in nontumor tissues; (b) p-Smad2 in low HOXB9 expressed HCC tissues; (c) p-Smad2 in moderate HOXB9 expressed HCC tissues; (d) p-Smad2 in high HOXB9 expressed HCC tissues; (e–f) negative control for nontumor tissues and HCC tissues, respectively. (B) Semiquantitative analysis of p-Smad2 by Immunohistochemistry in tissues from different HOXB9 expressed HCC tissues and nontumor. (C–D) Western blot was used to detect the expression of TGF-*β*1 signaling pathway (including Smad7, Smurf2, TGF-*β*1, and p-Smad2) in different HOXB9 expressed HCC tissues and nontumor. (E–F) Western blot was used to detect the expression of MAPK signaling pathway (including ERK1/2, p-38, and JNK pathway) in different HOXB9 expressed HCC tissues and nontumor. ^∗^*P* < 0.05 vs. nontumor.

We then explored the potential mechanisms underlying the clinical significance of HOXB9 in HCC. Due to the involvement of TGF-*β*1 signaling in HCC progression [17], we determined the expression of phosphorylated-Smad2 in HCC tissues. As illustrated in Figures 2(a) and 2(b), the expression of p-Smad2 in the HCC tissues is higher than those in the nontumor tissues, and its expression in the high HOXB9 expressed tissues is higher than those in the low HOXB9 expressed HCC tissues. Meanwhile, the results of Western blot revealed that the expression of HOBX9, as well as the markers of TGF-*β*1 signaling, including Smad7, Smurf2, TGF-*β*1, and p-Smad2, was highly expressed in HCC tissues compared with those in the nontumor tissues. Smad7, Smurf2, TGF-*β*1, and p-Smad2 protein expression was higher in the high HOXB9 group than those in the low HOXB9 group (Figures 2(c) and 2(d)). Induction of metastatic phenotype by TGF-*β*1 signaling in tumor needs alteration in additional signaling cascades including MAPK [18]; thus, we detected the status of MAPK signaling pathway in HCC tissues. We found that the expression of p-ERK was in sync with HOBX9 and TGF-*β*1, expressed higher in the high HOXB9 group than those in the low HOXB9 group and in the nontumor group (Figures 2(e) and 2(f)). However, there was no significant change in the levels of p38 and JNK1/2 signaling pathway between the HCC tissues and nontumor tissues.

**Figure 3:**
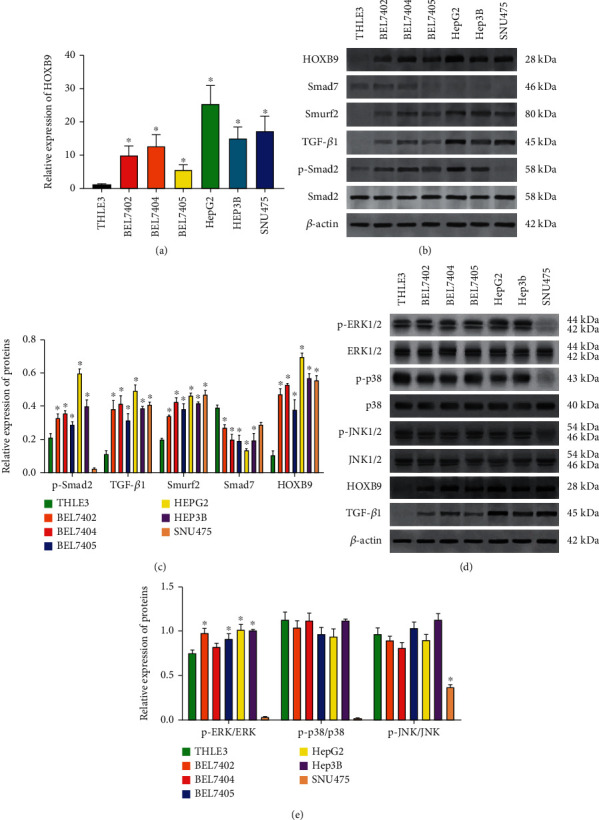
Expression prolife of HOBX9, TGF-*β*1 pathway and MAPK pathway in HCC cell lines. (a) RT-qPCR analysis of HOXB9 expression in immortalized human live cell (THLE3) and HCC cell lines (including BEL-7402, BEL-7404, BEL-7405, HepG2, Hep3B, and SNU475). (b–c) Western blot was used to detect the expression of TGF-*β*1 signaling pathway (including Smad7, Smurf2, TGF-*β*1, and p-Smad2) in THLE3 cells and HCC cell lines. (d–e) Western blot was used to detect the expression of MAPK signaling pathway (including ERK1/2, p-38, and JNK pathway) in THLE3 cells and HCC cell lines. ^∗^*P* < 0.05 vs. THLE3.

Subsequently, we analyzed the expression of HOBX9 in various HCC cell lines. RT-qPCR showed that HOBX9 was expressed at a low level in the normal human liver cells (THLE3 cells). The expression of HOBX9 mRNA was higher in all HCC cell lines when compared with THLE3 cells (Figure 3(a)). Equally, Western blot analysis showed that all HCC cell lines displayed higher HOBX9 protein levels compared to THLE3 cells. Moreover, except SNU475 cell, the expression of the markers of TGF-*β*1 signaling, including Smad7, Smurf2, TGF-*β*1, and p-Smad2, was highly expressed in HCC cell lines compared with those in THLE3 cells (Figures 3(b) and 3(c)). When referring to the MAPK signaling pathway, we found that the expression of p-ERK levels was significantly increased in HCC cell lines than those in the THLE3 cells (Figures 3(e) and 3(f)). However, there were no significant changes in the p38 and JNK1/2 signaling pathway between the HCC cell lines and THLE3 cells (Figures 3(d) and 3(e)). Taken together, the above data indicated a close relationship between HOBX9 and TGF-*β*1 and p-ERK signaling pathway in HCC.

### 3.3. HOXB9 Promotes Cell Proliferation, Migration and Invasion of HCC Cells

**Figure 4:**
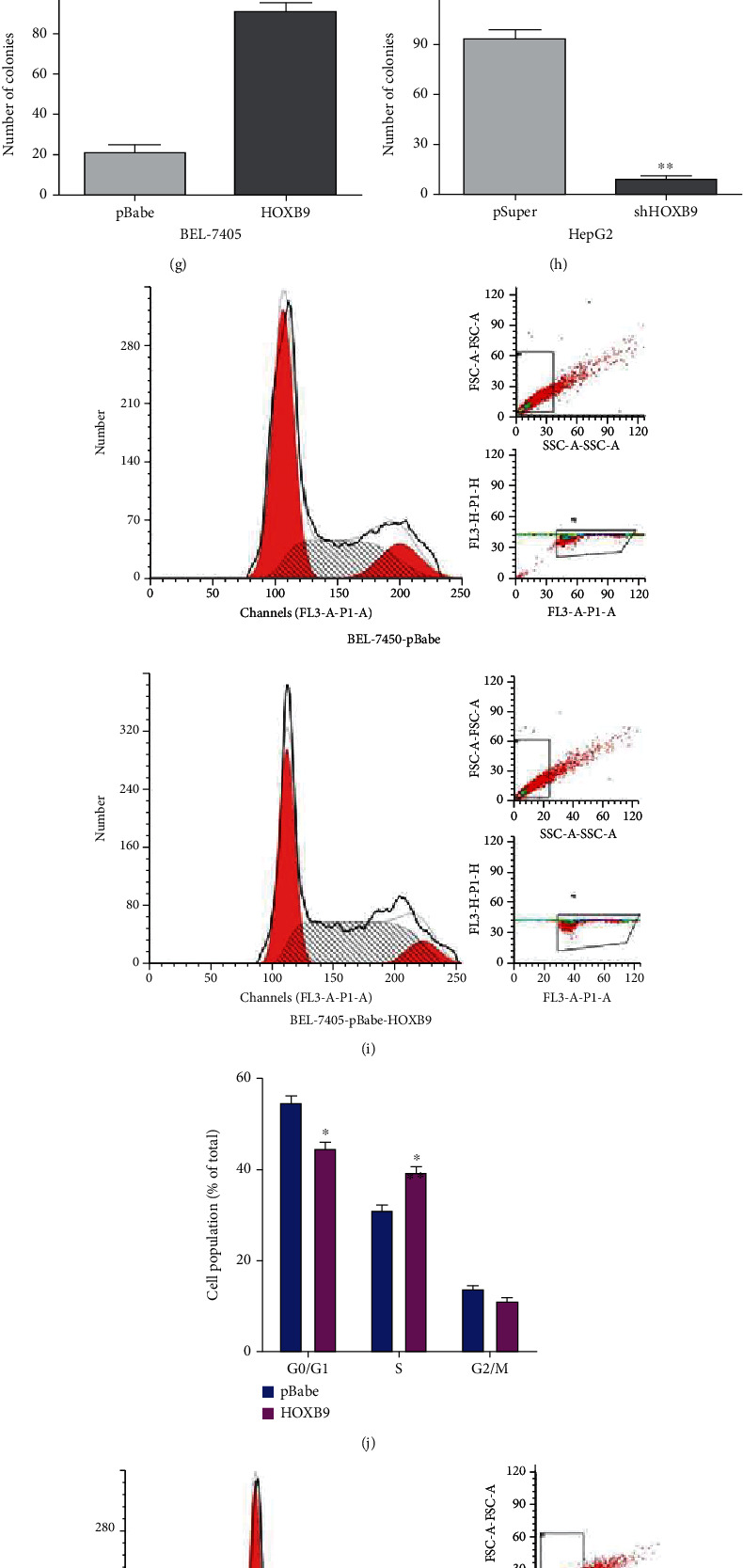
Effects of HOXB9 on cell proliferation and cell cycle, in vitro. (a–b) RT-qPCR and Western blot were used to detect HOXB9 expression in BEL-7405 cells transfected with pBabe or HOXB9, respectively. (c–d) RT-qPCR and Western blot were used to detect HOXB9 expression in HepG2 cells transfected with pSuper or shHOXB9, respectively. (e–f) BEL-7405 or HepG2 cell proliferation after the expression of HOXB9 was downregulated or upregulated, respectively, as assessed by MTT assay. (g) Colony formation assay was used to cell proliferation after overexpression of HOXB9 in BEL-7405 cells. (h) Colony formation assay was used to cell proliferation after knockdown of HOXB9 in HepG2 cells. (i–j) flow cytometry was used to determine the changes of cell cycle of BEL-7405-pBabe or BEL-7405-pBabe-HOXB9 cells. (k–l) flow cytometry was used to determine the changes of cell cycle of HepG2-pSuper or HepG2-shHOXB9 cells. ^∗^*P* < 0.05 vs. BEL-7405-pBabe; ^∗∗^*P* < 0.05 vs. HepG2-pSuper.

**Figure 5:**
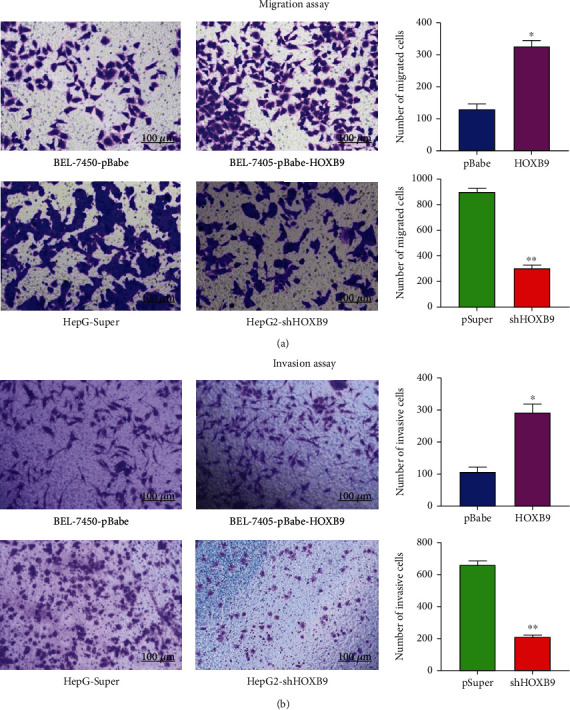
Effects of HOXB9 on cell migration and invasion, in vitro. (a) Transwell migration assay showed the effect of HOXB9 overexpression or knockdown on cell migrative ability in BEL-7405 and HepG2 cells. (b) Transwell invasion assay showed the effect of HOXB9 overexpression or knockdown on cell invasive ability in BEL-7405 and HepG2 cells. ^∗^*P* < 0.05 vs. BEL-7405-pBabe; ^∗∗^*P* < 0.05 vs. HepG2-pSuper.

To investigate the biological functions of HOXB9 in HCC, overexpression and knockdown of HOXB9 were performed in BEL-7405 and HepG2 cells, respectively, and the efficiency was verified by RT-qPCR and Western blot analysis. As shown in Figures 4(a) and 4(b), the expression of HOXB9 is marked increased in the HOXB9 group when compared with the pBabe group both at mRNA and protein levels. On the contrary, the expression of HOXB9 was decreased in the shHOXB9 group when compared with the pSuper group at mRNA and protein levels (Figures 4(c) and 4(d)). Subsequent CCK-8 assay showed that overexpression of HOXB9 enhanced the proliferative ability of BEL-7405 cells (Figure 4(e)), whereas inhibition of HOXB9 significantly inhibited cell proliferation of HepG2 cells (Figure 4(f)). Similarly, colony assay showed that overexpression of HOXB9 promoted cell growth in BEL-7405 cells (Figure 4(g)), and downregulation of HOXB9 suppressed cell growth in HepG2 cells (Figure 4(h)). Cell cycle analysis then suggested that overexpression of HOXB9 increased S phase arrest in BEL-7405 cells (Figures 4(i) and 4(j)), and downregulation of HOXB9 promoted the S phase transition in HepG2 cells (Figures 4(k) and 4(l)). Besides, Transwell migration assays reveled that upregulation of HOXB9 notably facilitated migration of BEL-7405 cells, while downregulation of HOXB9 suppressed migration of HepG2 cells (Figure 5(a)). Transwell invasion assays showed that upregulation of HOXB9 facilitated invasive ability of BEL-7405 cells, while downregulation of HOXB9 suppressed invasive ability of HepG2 cells (Figure 5(b)).

### 3.4. HOXB9 Definitely Promotes HCC Cell Growth, *In Vivo*

**Figure 6:**
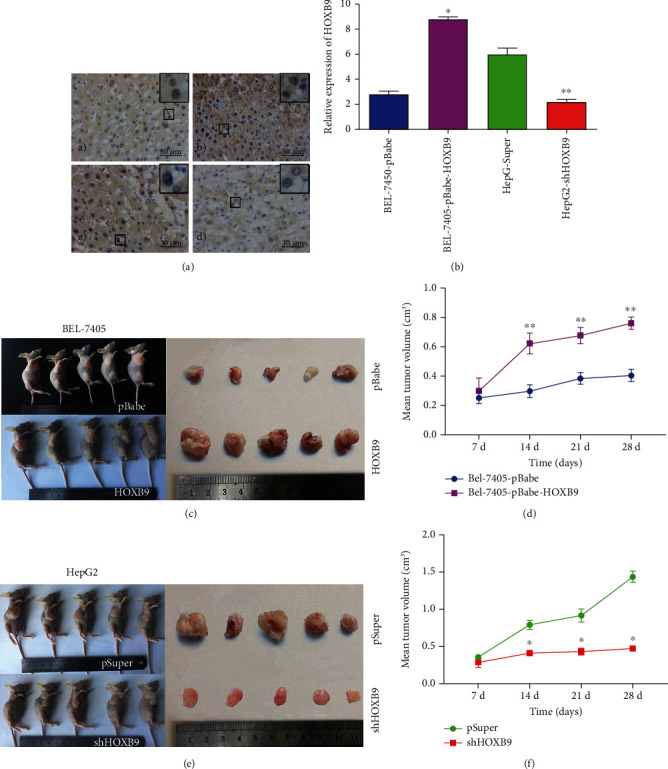
HOXB9 promotes cell growth HCC, *in vivo* (*n* = 5 per group). (A–B) The expression of HOXB9 was detected by immunohistochemistry in nodule from different group (200× magnification for the whole picture and 400× magnification for that at top right corner). (C) Images of subcutaneous xenograft tumors of BEL-7405-pBabe or BEL-7405-pBabe-HOXB9 cells and tumors were removed after 4 weeks of cultivation and photographed. (D) The changes of tumor volume of in the BEL-7405-pBabe or BEL-7405-pBabe-HOXB9 group were shown and compared. (E) Images of subcutaneous xenograft tumors of HepG2-pSuper or HepG2-shHOXB9 cells and tumors were removed after 4 weeks of cultivation and photographed. (F) The changes of tumor volume of in the HepG2-pSuper or HepG2-shHOXB9 group were shown and compared. ^∗^*P* < 0.05 vs. BEL-7405-pBabe; ^∗∗^*P* < 0.05 vs. HepG2-pSuper.

We then confirmed the function of HOXB9 in tumor growth, *in vivo*. IHC was used to confirm the changed HOXB9 expression in nude mice. As predicted, the expression of HOXB9 was increased in the BEL-7405-pBabe-HOXB9 group when compared with those in BEL-7405-pBabe group, while the expression of HOXB9 was significantly decreased in the HepG2-shHOXB9 group when compared with those in HepG2-Super group (Figures 6(a) and 6(b)). The size and weight of the tumors in the HOXB9 overexpressing group were markedly larger than its corresponding control group (Figure 6(c)), and the tumors in HOXB9 knockdown group were significantly lower than those in the control group (Figure 6(e)). The tumor growth model showed that overexpression of HOXB9 facilitated tumor growth (Figure 6(d)), whereas knockdown of HOXB9 notably inhibited tumor growth (Figure 6(f)).

### 3.5. Correlation of HOXB9 and TGF-*β*1 Pathway, *In Vivo*

**Figure 7:**
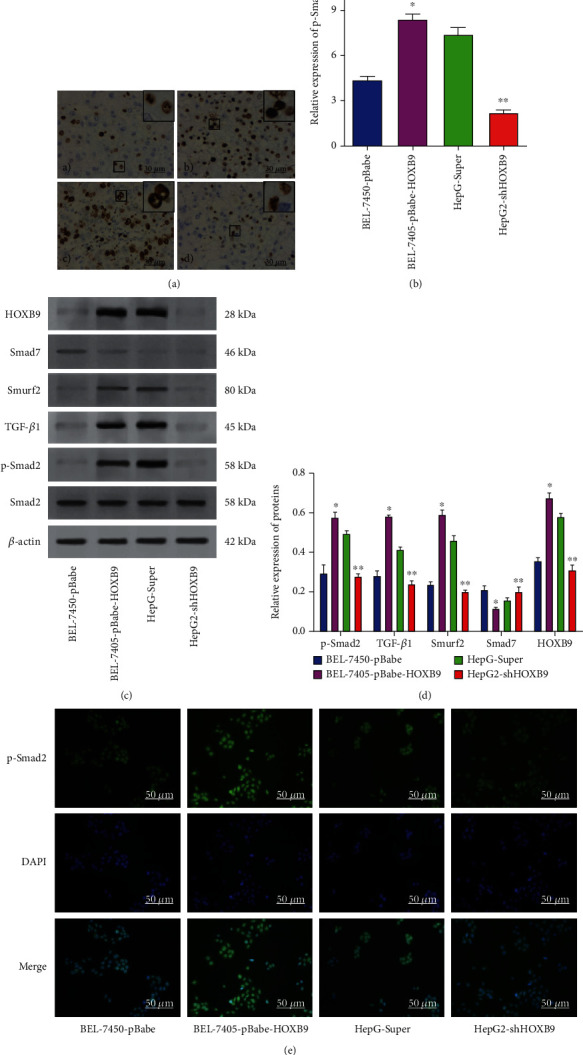
Effects of HOBX9 on TGF-*β*1 signaling pathway in the tissues from xenograft in nude mice. (A–B) Immunohistochemistry was used to reveal the effect of HOXB9 overexpression or knockdown on p-Smad2 expression in the tissues from xenograft in nude mice (200× magnification for the whole picture and 400× magnification for that at top right corner). (C–D) Western blot analysis was used to determine the changes of TGF-*β*1 signaling pathway (including Smad7, Smurf2, TGF-*β*1, and p-Smad2) in the tissues from xenograft in nude mice with HOXB9 overexpression or knockdown. (E) Immunofluorescence was performed to evaluate the effect of HOXB9 overexpression or knockdown on p-Smad2 expression in nucleus of HCC cells. ^∗^*P* < 0.05 vs. BEL-7405-pBabe group; ^∗∗^*P* < 0.05 vs. HepG-Super group.

Moreover, we measured the levels of TGF-*β*1 and MAPK signaling in xenograft model, and the results of IHC showed that the p-Smad2-staining score was significantly higher in the HOXB9 overexpression group than in the BEL-7405-pBabe group, and significantly lower in the HOXB9 knockdown group than in the HepG2-Super group (Figures 7(a) and 7(b)). Tumors and extracted total proteins were isolated to evaluate the changes of TGF-*β*1 and MAPK signaling. Consequently, Smurf2, TGF-*β*1, and p-Smad2 expression was apparently increased, while Smad7 expression was decreased in the HOXB9 overexpressing group compared with the BEL-7405-pBabe group. Smurf2, TGF-*β*1 and p-Smad2 expression was decreased, and Smad7 expression was increased in the HOXB9 knockdown group compared with the HepG2-Super group (Figures 7(c) and 7(d)). Moreover, the results of immunofluorescence found that overexpression of HOXB9 in BEL-7450 cells increased the fluorescence intensity of p-Smad2 in the nucleus, whereas knockdown of HOXB9 in HepG2 cells decreased the fluorescence intensity of p-Smad2 in the nucleus.

### 3.6. HOXB9 Influences Cell Proliferation, Migration and Invasion by Mediating the TGF-*β* Pathway

**Figure 8:**
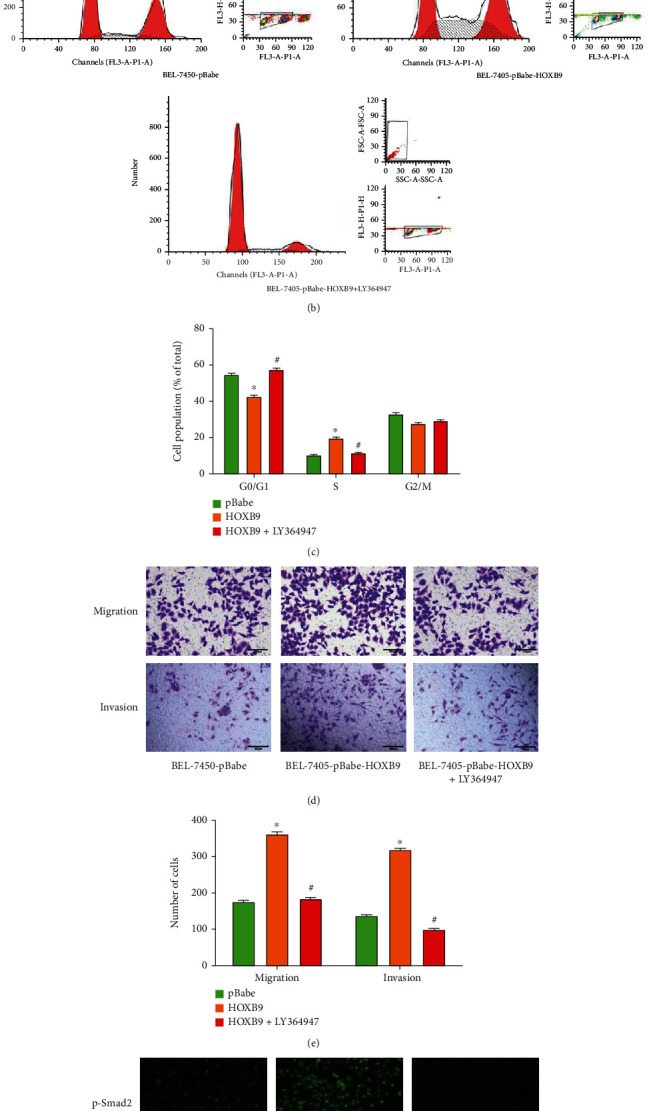
TGF-*β*1 signaling pathway participates in the role of HOXB9 in HCC cells. (a) MTT assay was used to analyze the cell proliferation of BEL-7405 cells, treated with pBabe, pBabe-HOXB9, or pBabe-HOXB9 and specific TGF-*β*1 inhibitor LY364947. (b–c) Cell cycle analysis of BEL-7405 cells treated with pBabe, pBabe-HOXB9, or pBabe-HOXB9 and LY364947. (d–e) Transwell assay was used to show the migrative and invasive ability of BEL-7405 cells treated with pBabe, pBabe-HOXB9, or pBabe-HOXB9 and LY364947. (f) Immunofluorescence was performed to evaluate the effect of HOXB9 and LY364947 on p-Smad2 expression in nucleus of BEL-7405 cells. ^∗^*P* < 0.05 vs. pBabe; #*P* < 0.05 vs. HOXB9.

To further support that the TGF-*β* signaling pathway was involved in the mechanism of HOXB9-mediated tumorigenesis, HCC cells were treated with TGF-*β* type I receptor kinase inhibitor, LY364947. As shown in Figure 8(a), BEL-7405 cells treated with LY364947 effectively reverse HOXB9 overexpression-enhanced cell viability. In addition, FCM analysis found that the treatment with LY364947 could abrogate HOXB9 overexpression-induced S phase arrest (Figures 8(b) and 8(c)). Subsequent Transwell analysis confirmed that LY364947 successfully reversed HOXB9 overexpression-enhanced cell migration and invasion (Figures 8(d) and 8(e)). Furthermore, the result of immunofluorescence indicated that fluorescence intensity of p-Smad2 was obviously impaired in BEL-7405 cells with upregulation of HOXB9 (Figure 8(f)). Taken together, these results indicated HOXB9 promoted HCC progression by mediating TGF-*β* signaling pathway.

## 4. Discussion

Our previous study had validated that overexpression of HOXB9 stimulated the proliferation of HCC cells, whereas the knockdown of HOXB9 produced an opposite effect [15]. Further, in the present study, we validated the role of HOXB9 in HCC progression and revealed that its action was dependent on TGF-b1/Smad2 and ERK1/2 signaling pathways. The roles of HOXB9 in tumorigenesis and disease progression seem to vary between different tumor types. Elevated HOXB9 was correlated with the advanced clinicopathological staging and identified as a prognostic marker in lung cancer [19]. In breast cancer, increased HOXB9 was reported to promote the progression and metastasis of the cancer cells [20]. On the contrary, decreased HOXB9 was considered as a factor for poor prognosis in colon adenocarcinoma and pancreatic cancer, as well as gastric cancer [21–23]. The above data indicated the complex roles and mechanisms underlying tumorigenesis and disease progression of HOXB9. In the current study, we validated the elevated HOXB9 expression both in HCC tissues and cell lines. Consistent with our study, a previous study by Chiba et al. has identified that HOXB9 was upregulated in HCC tissues and increased HOXB9 levels in HCC predicts poor overall survival, but a beneficial response to sorafenib [24]. Similarly, the present survival of patients with high HOXB9 expression was lower than the patients with low HOBX9 expression in our study. Moreover, Xiong et al. further found that ubiquitin-like protein FAT10 could regulate HOXB9 expression by modulating the *β*-catenin/TCF4 pathway, therefore promoting the invasion and metastasis of HCC [25]. They also identified that glucose-regulated protein 78 activated the Wnt/HOXB9 pathway to promote maturation of low-density lipoprotein receptor-related protein 6 and to regulate the invasion and metastasis of HCC [10]. The above data strongly indicated the regulatory role of HOBX9 in HCC. However, to date, how HOXB9 participates the progression of HCC remains unclear, which promotes us to conduct our study.

The association analysis between the degree of HOXB9 expression and clinicopathological characteristics revealed a significant correlation between HOXB9 expression and HCC tumor volume, suggesting that HOXB9 may facilitate the growth of HCC. Consistent with our previous study [15], HOXB9 promoted HCC cell proliferation and migration *in vitro*, as well as tumor growth *in vivo*. To elucidate the possible mechanisms by which HOXB9 regulates the malignant behavior of HCC cells, we also analyzed the relationship between HOXB9 and TGF-*β*1 and MAPKs signaling pathway, which are critical tumorigenic event contributing to hepatocarcinogenesis [26, 27]. By applying RT-qPCR, Western blot, and IHC, we found that HOXB9 was positively correlated with TGF-*β*1 signaling pathway Smads-related proteins (TGF-*β*1, p-Smad2, Smurf2, and Smad7) and non-Smads pathway MAPK-related proteins (p-ERK1/2). The above data suggested that HOXB9 may affect the expression of TGF-*β*1, which in turn activates p-Smad2 and p-ERK1/2.

Currently, the role of TGF-*β*1 signaling in HCC remains controversial. TGF-*β*1 pathway may play a tumor suppressor role in the early phases of HCC development [28], whereas promoting epithelial-mesenchymal transition in favor of tumor growth in the later phases [29]. Abnormal activation of the TGF-*β* signaling pathway in Hep3B, BEL-7404, and BEL-7402 cells [30] indicates that the activation of the TGF-*β*1 pathway is a common feature of HCC cells. We found that TGF-*β*1 pathway was significantly activated in high HOXB9 expressed tissue, which may be because the HCC patients enrolled was not in an early stage. Previous studies have shown that in gliomas, HOXB9 can directly bind to the promoter of TGF-*β*1 to induce TGF-*β*1 expression [31]. Also, HOXB9 is able to activate TGF-*β*1 signaling pathway to promote the invasive migration of tumor cells in oral squamous cell carcinoma [32]. More importantly, our previous study has identified that HOXB9 overexpression could promote TGF-*β*1-induced epithelial-mesenchymal transition of HCC cells [33]. Here, our *in vitro* experiments further found that inhibition of TGF-*β*1 pathway by specific inhibitors could effectively abolish HOXB9 overexpression induced cell malignant ability. The phosphorylation level of Smad2 was reduced, and nuclear transport was inhibited after co-administration of TGF-*β*1 pathway inhibitors. The above data indicated that TGF-*β*1 pathway was essential for the role of HOXB9 in the hepatocarcinogenesis.

Taken together, our study suggested that aberrantly high HOXB9 expression in HCC could promote the proliferation, invasion, and migration ability of HCC tumor cells by activating TGF-*β*1-mediated Smads and ERK1/2 signaling pathways.

## Figures and Tables

**Table 1:** HOXB9 expression in HCC and corresponding nontumor tissues.

Features	*n*	HOXB9 expression		*P* value
		High (*n* = 58)	Medium + low (*n* = 31)	
Age				0.399
<50	55	34	21	
≥50	34	24	10	
Gender				0.377
Male	68	46	22	
Female	21	12	9	
Histological differentiation				0.750
Well	9	5	4	
Moderate	47	32	15	
Poor	33	21	12	
Liver cirrhosis				0.900
Yes	41	27	14	
No	48	31	17	
Tumor size (cm)				0.028
<5	35	18	17	
≥5	54	40	14	
Metastasis				0.491
Yes	21	15	6	
No	68	43	25	
Recurrence				0.946
Yes	32	21	11	
No	57	37	20	
HBsAg status				0.766
Positive	76	50	26	
Negative	13	8	5	
Serum AFP (ng/ml)				0.605
<25	26	18	8	
≥25	63	40	23	

## Data Availability

The data used to support the findings of this study are available from the corresponding author upon request.
